# Abstract of Proceedings of the Buffalo Medical Association

**Published:** 1867-07

**Authors:** T. M. Johnson

**Affiliations:** Sec’y.


BUFFALO

### Medical and Surgical Journal.

VOL. VI. JULY, 1867. No. 12.

### Original Communications.

ART. I.—*Abstract of Proceedings of the Buffalo Medical Association.*

TUESDAY EVENING, June 4th, 1867.

The meeting was called to order at the usual hour by the President, Dr. Eastman. Members present—Drs. Eastman, Abbott, Rochester, White, Gould, Trowbridge, Diehl, Kamerling, Strong, Gay, Cronyn, Little, Miner, Greene, Ayer, Wetmore, J. R. Lothrop, Ring and Johnson. The minutes of the last meeting were read and adopted.

Dr. Trowbridge, member of the Committee on Revision of the Constitution and By-Laws, reported progress, and asked for more time. By vote of the Association the report was accepted and more time granted.

The President then read the following Inaugural Address:

Gentlemen of the Buffalo Medical Association:—Elected for the second time to preside over your deliberations, I have reluctantly consented to accept the position to which your partiality assigns me. Permit me to revert to the record of the proceedings of the "Buffalo Medical and Surgical Association" for April, 1856, in which the following minutes are found, viz:

"At the April meeting of the Buffalo Medical Association, no business of interest to the profession at large was transacted. The Association terminated its existence and re-organized as a chartered corporation under the title of the 'Buffalo Medical and Surgical Association.' The following officers, who are also Trustees of the property of the Association, were elected for the ensuing year:

Dr. Sandford Eastman, *President.*
" Austin Flint, *Vice President.*
" Sanford B. Hunt, *Secretary.*
" James M. Newman,* *Treasurer.*
" William Howell,* *Librarian.*"

Of these officers, the writer only, remains; two have removed to other cities, and two have died. The new organization at that time numbered 33 members, of whom 18 remain of our number, 11 having removed from the city, and 4 have died.

The number of members attending our regular meetings averaged twenty. Many valuable papers were presented by different members on various medical and surgical subjects; among which may be mentioned "The Diagnostic value of the Buffy Coat," by Prof. Austin Flint; "Hair Dyes," by Prof. Geo. Hadley; reports of sundry interesting cases of Diseases of Women, by Prof. White; Mulilocular Cysts and Trephining, by Prof. Hamilton; "Croup," by Prof. Rochester, and "Fistula in Ano," by Dr. P. H. Strong.

In June previous, a committee, consisting of Prof. Austin Flint, Drs. Geo. N. Burwell and C. C. F. Gay, was appointed to report upon Pneumonia, and three or four valuable reports were the result of their labors. These papers attracted the attention of the profession throughout the country, and gave the Buffalo Medical Association an enviable reputation, and made their proceedings sought for and studied. Similar labors will produce like results now; and I trust the custom of appointing an essayist for each meeting, so recently re-adopted, will be continued. If each member will attend the meetings regularly and furnish a tithe of what has interested him in the interval of our meetings, much information will be obtained that will be of value to some of us at no very distant day. The older members owe it to the younger to take the lead in reporting cases of interest, thereby guiding and directing them in the right way—in a way that will produce a permanent impression upon their inquiring minds. We are all studying the same grand lesson—all striving to accomplish the

---

* Deceased.

same great end; our whole life is one of thought and labor—mental no less than physical—and we all know how fatiguing and depressing the former is compared with the latter. Some in our profession have advanced farther than another, have turned more pages on the great book of nature, and have more thoroughly investigated the phenomena of disease and the remedies adequate to its relief. Whatever knowledge any of us may possess we should not hesitate in communicating to each other for the general good. While I have great respect for age and experience in the profession, I wish to remind our newly fledged members that age alone does not bring wisdom. Hence they are enjoined to contribute their quota in promoting the interest and value of our monthly meetings.

The prevention of disease should be, and is, the earnest desire of every true-minded physician, and this can be accomplished in no more efficient way than in giving to our patients and families such information as will best improve the sanitary condition of their houses and premises. If each one would take the trouble to do this—here a little and there a little—infinite good would result in the preservation of the public health and the prolonging of human life. We might thereby be doing injustice to our pockets, but we should have the more enduring satisfaction of doing good. A limb is sometimes so impaired by disease that life can only be saved by amputating the diseased part. This is nothing less than an acknowledgment by the surgeon of the impotency of remedies; a confession of his failure in curing disease. Of how much greater satisfaction and importance is it to so guide and instruct our patients how to live that the disease itself may be prevented.

During the past winter a committee was appointed to revise the fee-bill for medical and surgical services. A report will be presented at an early day, when the subject will claim your candid consideration. "The laborer is worthy of his hire," and this is eminently true of the faithful physician. No man performs so much labor gratuitously as he does, and no man deserves more reward from those able to compensate him for professional services. The poor we have with us always, and none of our number will decline alleviating their suffering and administering to their relief to the fullest extent of our strength and ability. This is known to the profession, but the public do not so understand it, or if so, they have a very singular way of manifesting it. Patent as this fact is, I have yet to learn of any of our number who is insensible to the proper appreciation of a generous fee; and while we should not be exorbitant in our charges, we should claim and receive a suitable return for services rendered. I bespeak for their report such action as the interests of the profession demand.

Gentlemen, we enter upon a new year with much to cheer and encourage us. The Association is free from debt. The wisdom of its founders has been thoroughly tested. The institution is of *age.* Let us vie with each other in endeavoring to advance its interests and reputation, so that we may transmit to our successors an inheritance worthy their possession.

A vote of thanks was tendered the President for his address and a copy requested for publication.

After which Dr. C. C. F. Gay read the following paper:

*Some hints as to how the Surgeon should manage fractured bones; how he should manage his patient, and how the Surgeon should himself manage.*

Mr. President and gentlemen of the Association, unlike other departments of surgery, this opens up a field where the same amount of labor, patience, tact and skill do not, as in the domain of operative surgery, reward the laborer with a corresponding amount of *eclat.* Best efforts are not rewarded by corresponding approbation, but when bringing to his work, skill equal to that brought to bear in the other departments of surgery the surgeon's reward oftentimes consists in the displeasure of his patients and their friends, who, impelled by a species of innate depravity, attempt to appropriate the surgeon's capital, gratis, refusing payment for services on the ground of neglect or unskillfulness, proceed to mulct the attendant in damages, and legal proceedings are known sometimes to have been instigated by officious friends for the purpose of ridding themselves of the payment of an honest debt.

For the faithful cultivation of this field of surgical science, for labors directed toward the protection of the surgeon against suits for mal-practice and for advanced and progressive views upon surgical pathology, we are more indebted to the labors of Dr.

Hamilton than to any other American surgeon, and the members of this Association who were for so many years connected with him in these monthly séances, take pleasure in the acknowledgment of his worth and the recognition of his work as authoritative.

But, gentlemen, disclaiming any personal merits, it is competent to remark, that it was not reserved for one mind, however comprehensive, unaided and alone, during a single life time, to bring order out of chaos, make the rough and rugged places smooth, or by the touch of his magic wand bring out the gushing and sparkling waters, or in short, to bring to perfection and to an exact science that which is encumbered with so much that is imperfect, crude and shapeless. It may have been reserved for lesser minds to contribute lesser things. If in addition to the enlightened direction of medical and surgical teachings, the observations and studies of two decades have not taught us anything—if some old views have not been discarded, and new views when they have been tried and proved to be true views, been received, then have the labors of these years been of no service and the profession practiced in vain.

In indicating some hints how the surgeon should manage broken bones, the writer would rely chiefly upon his own experience and observation, growing out of his own and others' practice.

Authority in surgery has so long exercised such powerful influence upon the practitioner as to be detrimental to the best interests of science. It requires boldness and courage to deviate from authority; such departure or deviation is misconstrued into disrespect, or obstinacy, or ignorance; far be it from me to undervalue or underestimate authority in medicine or surgery. I yield to no one in my reverence to it and for it, but if we hope for progress in our art, and are not content that others do our thinking for us, we must sometimes become amenable to the charge of discourtesy and disrespect.

It is independent thought that we most need; a frank and clear presentation of those thoughts and a respectful hearing and reception of them, even though they do not emanate from those who have been regarded as authority. In this way will the combined and united labors of individuals be productive of good fruit.

In the management of fractures it is hardly necessary to say before this enlightened audience, that a full and correct knowledge of the surgical pathology of the reparative process, is an indispensable prerequisite to intelligent management; following this, the more genius and *tact* the surgeon possesses the better it will be both for himself and his patient.

I do not propose to occupy your time in discussing the pathology of the reparative process of broken bone; it is sufficient to say that a very great stride from error towards truth was that step taken by Paget, fifteen or twenty years since, when he stepped out of the beaten track, ignoring the theory long since laid down by Dupuytren, and establishing a theory of his own so much more rational, and not only rational, but a theory proved to be well founded by well attested experimentation and by well grounded observation.

If you would pardon an apparent digression, I would here pause for a moment, to pay a passing tribute to the genius of Hamilton, who, with Paget, is equally entitled to the honor and fame of establishing the new theory of the reparative process, arriving, as he did, at nearly the same conclusions, consummating his labors and publishing the results to the world about the same time.

The labor of these two men, directed in the same channel, toward the same object, giving results to the world almost simultaneously, has been the means of revolutionizing the management of fractures, or if this be too strongly stated to suit the fastidious, I will modify it by stating simply that the treatment of fractures based upon the knowledge imparted to the world by Paget and Hamilton, becomes rational and intelligent.

The surgeon has not to wait for the ninth day provisional callus of Dupuytren to serve as nature's splint, for he knows that when the fragments of bones are properly adjusted, especially when the fracture is near the extremity of long bones that union will occur with no more deformity, when no splints are used, than will occur when they are used; that splints are not so indispensable as formerlly supposed, and that they may be *laid aside much earlier* than we have been taught by the highest authority to believe.

When about to treat any special fracture, the first question of course which arises relates to diagnosis; and here, I believe, the most skillful surgeon may blunder, if he should allow himself to be hurried, and should not exercise proper care and attention in his manipulations. But the blunders are exceptional only—none ever need occur; but right here, at the threshhold of right management, *tact* is brought into requisition. I have seen patients suffer all the pains and torments of the inquisition, while submitting with commendable fortitude to rough and misdirected manipulations. I quote the language of a well known author, who says:

"It is difficult to state the precise manner in which the surgeon ought to proceed. Much will depend upon the circumstances of the case, something upon one's natural tact, and upon the amount of experience, but more I think upon natural kindness of heart and social education. The man of refinement and sensibility will know instinctively how to proceed, and needs no instruction. They who lack these qualities can never learn, and it would be quite useless to undertake to teach them. I sincerely wish such men as these latter would find some more suitable employment than the practice of a humane art."

With little experience and a good deal of tact, I am confident the majority of fractures may be diagnosed and dressed without inflicting much pain upon the patient, and the work accomplished in little time.

After the proper adjustment of the fractured bones to their normal position, he who possesses most genius for improvising the necessary apparel, then and there upon the spot, making his splints of the rude material at hand, will be found, I apprehend, most competent to adjust his apparatus. The more simple the apparatus the better, and a splint made and adapted to any special fracture is far better than the adaptation of a special fracture to a carved splint made to suit a supposed case.

The most important question now to determine is, the *length of time* the splints should be continued in use. Your time would not be lost if an entire paper, written by some competent person, should be devoted to this single topic. At this point in the discussion I find that the views of the highest authority and the views of the humble writer become divergent. All the care, precaution and tact possible, dwindle into nothingness in comparison with the right decision of the surgeon at this point, in determining the length of time for the use of splints. I do firmly believe that more injury is done by long-continued use of splints—by their use beyond their actual necessity—than by erring in the opposite direction. These views, which I assume to be true views in the management of all fractures, are manifestly true beyond cavil, when made applicable to fractures near joints.

The settlement of the question of the longer or shorter continuation of surgical appliances, in a legal point of view, is important. With this question unsettled, as we must concede it to be, it would prejudice a jury greatly to the detriment of a defendant in court, for the witness to swear that splints were removed in case of any fracture in ten days, or two weeks, while defendant's case could not at all be compromised should medical or other witness testify that splints were used for three or four months; use of splints almost any length of time, however long, is never prejudicial to the case.

It is no new thing for a medical witness to testify that deformity after fracture, is perhaps partially owing to the fact that splints were removed too soon. Who has ever thought to testify that in case of fracture near a joint, causing temporary or permanent anchylosis, that splints were not laid aside soon enough? It seems to be impressed upon the minds of judge, jury and witnesses, that the long-continued use of splints is beneficial, and that early to dispense with their use is detrimental.

In the case of Ostram vs. Kempson, cause tried before Hon. I. A. Verplanck, for mal-practice, in March, 1866, a verdict was rendered for $300 damages. This was a supposed case of Colles' fracture occurring in the person of a female, aged 64 years. The injury happened in November or December, 1863; suit brought March, 1866, a little more than two years subsequent to date of injury. On the trial the daughter of Mrs. Ostram testifies that "her mother first got use of her fingers three or four weeks ago; up to that time had no power over them; it was ten months or a year after injury before she was able to bring thumb and finger together, but had no strength; she can now clasp her hand, but can hold no weight," etc.

The defendant, Dr. Kempson, testifies, that "he removed the splints in *six weeks;* there was then a perfect state of arm, except a little deformity." One surgeon testified that, "in patient of Mrs. Ostram's age, arm should be kept in splints from *forty* to *sixty* days."

Now I take not the slightest exception to the merit of the testimony of these physicians, because the views therein expressed are in accordance with established custom, and are supported by authority, but I do take exception to the authority, and decidedly differ therefrom. I incline strongly to the belief that had the splints been removed in two weeks in the place of being used six weeks that Mrs. Ostram's daughter would have been able to testify that her mother was able to use her fingers much earlier, that strength of hand would have returned much sooner, and that the deformity would have been no greater.

But where may the surgeon be found of sufficient boldness and independence to strike hands with authority and testify that in case of Mrs. Ostram, splints should have been removed in two weeks? If such medical witness can be found so to testify, he would testify to that which is true—perform an act which would redound to his own credit—perform valuable service for the profression, and do a most humane act for the unfortunate patient.

I do not, gentlemen, speak in this manner of things I do not know, but I do thus speak of things that are true, of what I do know, have seen and believe; authority to the contrary notwithstanding. To enforce these views, were it necessary, examples could be cited, cases given of Colles' fracture, and fracture of the lower extremity of the fibula and internal malleolus—fractures wherein almost invariably the injury to the joint becomes paramount to the fracture, in which cases splints were removed in ten days, with result equal to any treated by longer use of splints, and perhaps far better result. But this paper will be of sufficient length and your patience sufficiently taxed without encumbering it with cases.

In fracture of lower extremity of fibula and internal malleolus, as with the fibula fractured with luxation of tibia, Hamilton says: "As a general rule the dressings ought to be wholly laid aside by the end of the third or fourth week." After this date he regards the use of splints and bandanges pernicious.

Conscious as I am of the danger of differing with so high authority, appearing not unlike presumption to attempt it, truth, nevertheless, requires of me to say, if my own experience and observation have taught me anything, they have taught me that splints should be wholly laid aside at the end of ten days, if the patient be either in middle life or approaching old age; and why? The Doctor, I think, clearly states the reason; because of the stiffness of the ankle-joint. This stiffness, he says, in three of his reported cases, remained after one year, and in one case two years, etc. It is this stiffness as well as deformity that the surgeon desires to avoid. When the bones, in this injury, are properly adjusted, they will remain in position with rest at the expiration of eight or ten days without any support from splints or bandages.

One other exception only do I wish, at this time to take, to what I regard the highest authority in this country. It has reference to the time when broken bones should be reduced. All will agree that broken bones should be reduced as soon as possible. But when once reduced, do they always remain so? Hamilton says, "when the injury is recent the muscles offer less resistance, their resistance being increased after a time not only by the reaction which ensues upon the shock, but also by actual adhesion between their fibres."

I think this statement amenable to criticism. Observation teaches, I think, that the pain, irritability, and the emotion of fear following immediately upon the receipt of injury, causes *then* greater muscular resistance than at any subsequent period.—Therefore, in the management of oblique fractures, remote from joints, equally with fractures near joints, good surgery requires at first loose and temporary dressings, and when swelling, muscular resistance and irritability subside, permanent dressings.

You will note that I have had in mind chiefly those fractures occurring *near joints.* I should be glad to be able to say that I had read Smith upon the topic under immediate discussion, but am sorry to say that I have never availed myself of the benefits to be derived from perusal of his able work, and consequently am entirely unacquainted with him, and know not what his views are.

In the management of fractures remote from joints, I have nothing to say, further than that I do not object to a longer use of splints than in fractures contiguous to joints. Successful management of this branch of the surgeon's art is somewhat intuitive and dependent upon native genius.

If not of the last importance, it certainly is not of the least importance for the surgeon to know how to manage his patient. Little things and "trifles though light as air," in the aggregate become potent, and applied to the practice of our art, give symmetry and strength to the whole. Medical minds bend reluctantly toward the smaller things that obtrude themselves along the route of research and labor. To neglect these trifles is to commit wrong. Our authors sacrifice the little things to the weightier matters of theory. Hope hath trodden upon the heels of despair and victory wrung from defeat, by the mere observation of the movements of a spider spinning its web. Laws of gravitation became established by witnessing the fall of an apple from its parent stem to earth. Despise, then, not the day of small things.

The surgeon's duties do not end with the broken limb mended, nor begin with the broken fragments adjusted. He should state to his patient frankly in the outset what to expect, and to what submit. His patient should be informed that his broken limb will be shortened, deformed, etc. Should the patient be dissatisfied with the prognosis, then turn him over to the tender mercies of some one who will promise better results. Should your services be accepted upon conditions stated, there will be no excuse in the patient failing to liquidate his obligations to you and no cause for complaint.

Beside the proper management of the broken bones and the right management of the patient, the surgeon has in charge also the management of himself. I have briefly indicated how he should *himself* manage, and it is only reserved to hint how he should conduct himself. Self-control is a virtue. It is good to know one's self, and if this be not enough to know, as the poet claims it is, then seek to be satisfied by knowing how to control thyself. However provocative the acts of patient and friends, which are often beyond endurance, however great the causes, which are numerous and ever present, the surgeon should so control himself that his own self-respect and the dignity of his vocation should not be lowered in tone by the exhibition of any ill temper.

Should there be refusal on your part to dress a limb for an irresponsible person, it may be done with decorum and without any exhibition of ill temper or in the least becoming obnoxious to such charge, especially when no *charge* is to be made. And right at this point I approach a delicate and debatable subject, but one relevant to the surgeon's management of himself, and you may, and doubtless will, dissent from the views of the writer, and perchance hold him obnoxious to the charge of inhumanity. I have learned to believe that the kindest and most humane acts of men are often seized upon by the ungrateful beneficiary, and turned upon the benefactor with relentless hate, and used as a two-edged sword, piercing to the dividing asunder of the joints and the marrow.

A few installments of the subjoined experience will, I think, amply teach the most humane surgeon how to manage himself, and how *himself* to manage under like circumstance.

Once upon a time, not very remote, I had occasion to congratulate myself upon the result of treatment of a Colles' fracture. Patient irresponsible; his employer, a man skilled in the manufacture of car-wheels and rolling stock machinery, becoming responsible for payment in full. Three months after date of injury, on presentation of bill, I was met with the impolite rebuff that if "my work had been well done, the man would have been at his work a long time before and have earned enough to have enabled him to pay his own bill." Thanking this man for the exhibition which he had given me of his ill-breeding and ignorance, I departed from his august presence. This man afterwards attempted to trump up sufficient evidence to convict of mal-practice; accordingly he sent this patient to a number of physicians to obtain their professional opinions. But to the honor of the medical profession of Buffalo, be it said, no sympathy or encouragement was extorted from a single member.

It was once my misfortune to be called hard-hearted, because of refusal to dress a broken arm. The employer stepped forward and became surety, took patient to his own house, providing him with meat, drink and bedding. When able to leave, this ungrateful wretch quitted the country for the Queen's dominions, taking with him my splints, and leaving me to liquidate his board and lodging account, which I partially did, by deducting one-half the bill for surgical services rendered.

I therefore appeal to the most humane surgeon—to laymen and to an enlightened community to endorse me, while I briefly indicate how the surgeon should himself manage, when in the legitimate practice of his profession under difficulties of this nature. I think it may be laid down as a rule of action to refuse to dress a broken bone for an irresponsible person unless such person be willing to bind himself in writing never to carry his case into court, or unless some reliable individual will agree then and there to stand between the surgeon and suit for mal-practice. The only exception to this rule is that made in favor of widows and orphans. No man, I apprehend, is willing, voluntarily, to place himself in position to defend himself against suit for mal-practice, when his services, if rendered, are beyond hope of reward. No man is willing to assume such relation with the chances all in his favor of being mulcted in damages, and no surgeon should be forced by popular sentiment to serve the community upon any such unequal terms. But when holding himself up and offering his services to the community as competent to discharge with fidelity and ability the duties of his office, I hold that the surgeon is necessarily forced, if he be not willing, to perform all service to patients when called upon, provided he is unquestionably to receive remuneration for the same.

Thus, gentlemen, have I endeavored in as few words as possible, to put forth views upon the topics discussed, which to me seem to be true views. This paper does not aspire to the dignity of a scientific "dissertation upon a designated subject." The writer has only sought to give expression to views, crude and undigested; such, in a word, as have suggested themselves to his mind during haste in writing them out, to throw out a few practical hints upon a somewhat practical subject, and to group together the neglected trifles, of which nothing is read or said, and as little written. If I could hope that the contents of this hastily written paper would meet your approbation, then should I feel myself abundantly rewarded for the little labor bestowed upon it.

Thanking you, gentlemen, for your kindness in making me the recipient of many courtesies and favors in times past, my obligations are again renewed for the kind and cordial invitation extended to me to prepare a paper for the Association, and for the attention given and interest manifested during its reading.

Dr. White remarked that he hoped Dr. Gay would explain to the Association the precise condition of the fractured bone at the tenth day.

Dr. Gay replied that he did not intend to go into an explanation of that subject, and would refer inquirers to the books.

Dr. White said, I cannot entirely agree with Dr. Gay in regard to the removal of splints. In my younger days I read surgery carefully, and had to defend suits brought for mal-practice, and have always used my best endeavors to vindicate the profession. With my understanding of the pathology and repair of fractured bones, it seems to me that if we are to remove the splints at the end of ten days there is no necessity for putting them on at all. I am much pleased with the paper in many respects, but must disagree with the Doctor upon this point.

Dr. Rochester said, perhaps Dr. Gay will inform us upon this subject. I fear that if the paper goes out as a part of the proceedings of this Association, and is read by young medical men, it may do harm, and be the cause of many suits of mal-practice, and be detrimental to this Association.

Dr. Strong said that he must demur to the idea that the paper would be detrimental to this Association. The Association is not responsible for papers read here, or for all the ideas advanced. The paper, as read, is not vouched for by us, and we are therefore not responsible. The view taken by Dr. Gay is extreme; it is Dr. Gay against the world. I cannot see the propriety of placing the splints on for ten days. They may as well be left off entirely.

Dr. Miner said that, as he was not present at the reading of the first part of the paper, it was fair to infer that he did not understand the full scope of the author's meaning. If the idea was intended, that in fracture extending into articulating surfaces of joints, splints were often prejudicial, if continued longer than to allow subsidence of the early inflammatory action, he could quite agree with the author; or if it was the intention to say that in fracture extending thus into joints no splints were necessary, and that *motion* was to be maintained rather than rest—motion of the joint, not of the fracture — the author's views would appear quite consistent with the best mode of practice. If the paper favors the use of extension only, and regards the extended muscles as adequate support to fractured bones, especially after the first symptoms have subsided, it will appear also as quite consistent. That splints, which were at any time requisite, can be safely dispensed with the tenth day, appears quite inconsistent with the usual condition of the parts in fracture and of our notions of the objects to be gained by their use. The time of union of bones varies greatly, and no invariable period can be given. In children, twenty-one days may suffice, but adults require much more, and bones which *appear* united by bony union, if left unprotected will often bend, even after the third month. Such instances are not uncommon; they come under the observation of all surgeons. The general idea that fractured bones are often subjected to, too much confinement must certainly be correct, but that they do not require support only for a few days requires qualification and explanation.

One other suggestion of the paper should not pass unnoticed. The author advises surgeons not to dress fractures for irresponsible persons unless some one becomes responsible for the payment of their bills. It is very true that surgeons receive very small compensation for the care and responsibility of such service, and anything which will change the condition of things in this respect, is desirable, but I am in the habit of going everywhere, and dressing everything, when called, and it seems to me a matter of duty and of necessity. I rarely suffer from unhesitating attendance in such cases, and though I dress and treat fractures, often without compensation, it is not generally of the class where a surgeon would once think of asking any one to be responsible. It is remarkable, considering the class of people who most frequently receive fracture, how very few cases but are kindly considered by some one. The employers in manufactories or the benevolent in neighborhoods almost invariably share with the surgeon in the care of the unfortunate, who are poor; and I had rather take the chances of pay, without any special guaranties, than with. When payment has been guarantied to me, I have generally in the end received nothing; but I have never in a single instance asked any one to become legally responsible. So much of the paper as represents the great responsibility, both professionally and legally, of dressing fractures, cannot be too heartily approved, and any action which can relieve, or compensate for it, should meet most hearty approval. That fractures should be immediately adjusted, without waiting for the inflammation and swelling to abate, cannot now be questioned, though there are practitioners who adhere to the old views of surgeons, and think they cannot be put up in "permanent dressings" as they call them, until the fourth or fifth day.

There are several other topics suggested by the paper which are important and entitled to consideration, but I will leave the discussion of them for others.

Dr. Cronyn said, that there were many things in the paper just read of an ethical character worth consideration and observance in many cases; but the surgical teaching of the paper was open to many and grave objections. The author asserts boldly "that many of the deformities after fracture are due to the *too* long continued application of splints; that in the greater number of cases ten days would be quite long enough to continue any such application," etc., etc. This will do very well for fracture of the fibula or of the internal malleolus or under such circumstances where little or no surgical support is needed, and the patient of such an age and disposition as to subject himself voluntarily to the required rest; but what would the author do with fracture of the femur near the lesser trochanter, more especially if it happened to be oblique? Could he take off his splints in ten days and have a good result? It requires but a moment's recollection of the powerful action of the psoas and illiaca muscles in this fracture, and the too well known difficulty of, under any circumstances whatever, having a good limb. It is unquestionably proper to remove splints from fractures in the vicinity of joints as early as possible, but as Dr. White has said, if they can be set aside as early as ten days, there would seem very little need for their use at all, and as the author has not advanced any new theory of the reparative process by which he could prove the soundness of his surgery, it would seem a dereliction of duty should this Association allow his teaching to pass without discussion or comment, nor would it be any compliment to the worthy author to so permit it.

With regard to the theory of provisional callus, (Dr. Hamilton's,) he does not quite represent that learned gentleman's opionion, for Dr. H. does admit that under certain circumstances such provision is made, and it would seem that the various experiments of Hunter, Dupuytren, and others, as well as the more recent teaching of Paget, confirm beyond question, the almost constant occurrence of ensheathing or provisional callus. The exceptions to this rule are well known to most surgeons of the present day, of any experience at all.

Dr. White said, I regard it proper to dress a fracture as early as possible. I believe it as necessary to keep on the splints the second ten days as the first ten. I do not think it complimentary to say that a paper, as valuable as the one read, should be disowned by the Association. There is justice in holding the Association, to a certain degree, responsible for the assertions or ideas of a member if the assertions or ideas be not controverted by the members present at its reading.

Dr. Gay said, so much time has been spent that I am unwilling to take time to fully discuss this matter, and will only say that fractures, remote from joints, were not alluded to. It was not intended to give the mode of treating them. In regard to bad results from fractures, we often hear it said that the splints were removed too soon. I believe that they are oftener removed too late. I believe that there is no provisional callus. I believe that there is sufficient effusion within a few hours after a fracture to answer the purpose of the reparative process of the broken bone. This effusion or secretion does not extend beyond the broken surface of the bone.

Dr. Ring said, I believe in provisional callus; once saw a colt that died ten days after it had fractured one of its legs. At death the provisional callus was well marked.

Dr. Eastman said, three years ago a patient with a fractured tibia and fibula died from a cause remote from the fracture. I examined the fractured bones sixteen days after the fracture, and found no provisional callus; found only an effusion of a gluey substance. There was no hardness between the ends of the bones. There was no provisional callus in this case; the union was not complete. Every good surgeon knows that in all cases of fracture near a joint the dressings should only be put on sufficiently firm to retain the fragments in position and prevent motion at the point of fracture. It is improper to retain dressing as long in this case as in cases remote from joints, because of the danger of anchylosis of the joint.

Dr. Lothrop understood Dr. Hamilton's belief to be, that the amount of material furnished for what is called the provisional callus, depends very much upon the ability of the tissues to provide it. In some situations more is furnished than in others. In the flat bones there is no formation of callus, while in the long bones it is usually abundant. This has been held to be a wise provision of nature, that callus should be furnished where most needed, i. e. where displacement is most likely to occur and where broken bones most require support. It is familiar enough to all that in certain situations bunches soon form at the point of fracture, caused by plastic material thrown out; but the amount of it depends very much upon the situation and the nature of the tissues. In situations where the tissues are abundant and vascular the material is abundant; but in thin and not very vascular tissues there is little or none. It arises then, in fact, from the re-action of the tissues, and not from any wise precaution on the part of nature.

Speaking from his own experience he had never met with a case in which the formation of provisional callus could be demonstrated. He could, however, call to mind one case, in his own experience, in which he had an opportunity to examine a broken bone some time after the fracture. In that case there was nothing to answer to what is thought to exist where callus is found. A man severely injured in the knee-joint, by railroad accident, came under his care at the Buffalo General Hospital. At the time, the fracture which was near the joint, was not detected, as there was no displacement. After a trial it was found that the knee-joint could not be saved, and amputation of the femur was performed one month after the injury. Upon examination a fracture of the femur was found at a point an inch and a half above the joint, but no displacement had occurred, and a partial union had apparently taken place. Yet there was nothing like a provisional callus. Some osseous material was found in the tissues at the seat of fracture, but nothing like callus. There was no difficulty in removing all the tissues from the bone. Osseous material is undoubtedly often found in the neighborhood of injuries to bone, especially in deep tissues. But it appears to take place in accordance with a pathological law, known as the law of analogous formation, rather than for the purpose of furnishing callus. By that law a formative action is exerted upon plastic material by the tissue into or upon which it is effused. If in the neighborhood of bone it takes on an osseous character. Thus what has been called a provisional callus, and thought to be needed for the support of broken bones, explained in this way, seems rather a result of two causes, first the re-action of the tissues providing plastic material, and secondly the developmental character given it by the influence of the part in the vicinity.

The President said, that some interesting operations had recently been made in the city for removal of ovarian tumors by Dr. White and Dr. Miner, and reports of the cases would be interesting to the Society.

Dr. James P. White reported two cases of ovariotomy in which he had operated since our last meeting. His object in referring to them was not because the operation is new or of doubtful utility. For more than fifteen years all, familiar with my teaching and practice are aware that I have regarded its feasibility as well settled as the amputation of the thigh. It is in fact, now, a common operation in England and this country. My object in relating these cases is to speak of the novelties in the operation as now performed. During the last year have had repeated opportunities to witness the operations of Baker Brown, Spencer Wells, and other distinguished men of the old world. My operation is an eclectic one, combining many of the features of the different London opertors and some things which originated with myself. I regard the cauterization of the pedicle and its return into the abdomen, in all suitable cases, of great importance. The hot iron should not be used when heated to a white heat, but at a red heat, and if judiciously and carefully used, seldom fails. I prepare the patient for operation by placing her upon a firm cot bed, upon which she is to remain after the operation, and dressed in her usual bed or night clothing. Before proceeding to operate I place upon her, next the surface, a rubber sheet with a hole in the centre, large enough to expose the surface sufficiently to make the operation; the edges of the hole should be made fast to the surface by adhesive plaster. This rubber sheet should be large enough to project beyond the edges of the bed. The utility of this is, that it perfectly protects the patient, her clothing, and the bed clothing during the operation, and also prevents exposure of the person. The patient is then placed in the centre of the bed and upon her back.

The temperature of the operating room shouid be 95 or 96 degrees. Before commeneing to operate I have fifteen or twenty small, fine sponges, made soft and pliable by having been soaked in warm water for at least twelve hours before the operation. While in use, during the operation, these sponges should be dipped in water of at least 95 degrees, into which has been put a little carbolic acid. I use no cold water during the operation, and aim to have everything coming in contact with the parts operated upon of the same temperature as that of the body, and have all my assistants in the operation wash their hands in warm water, into which carbolic acid has been put. The deep sutures, those which extend down to the peritoneum are of silver wire. The superficial sutures are of silk. The silk sutures are inserted from within outwards, each silk having a needle at each end and inserted intermediate between the silver sutures. After the sutures are adjusted I place adhesive straps two or three inches wide entirely around the body, so as to completely bandage the whole abdomen, from the ensiform cartilage to trochanter. The utility of this is, that it supports, steadies and strengthens the parts and prevents irritation. I use a large trochar with a canula three-fourths of an inch in diameter. If the tumor is multilocular I eviscerate. I give nutriment, usually beef essence, just before and just after operation. Give stimulants, if necessary. Always give an anodyne before operating. The urinary bladder should be evacuated before the operation, and it should also be evacuated at least three or four times a day during convalescence. The after treatment according to indications—no definite rules.

The first case that I report is that of the wife of a respectable practitioner of medicine, residing in Michigan, aged 31. Diagnosis doubtful; abdomen large. Made the operation on the 8th day of May, 1867. Present—Profs. Rochester and Eastman, and Drs. Wetmore, Abbott, Daggett and Diehl. Chloroform by Prof. Rochester. Exploration confirmed diagnosis. The tumor was removed and pedicle cauterized and returned into abdomen. Time occupied, 25 minutes. Tumor and contents weighed twenty-five lbs. Wound closed by sutures as described. No untoward symptoms. Silk sutures removed 6th day; bowels moved on the 8th; menstruated on the 16th day, and on the 21st the husband left for home, convalescence completely established, patient moving about freely, and now quite well.

*Second Case.*—Mrs. ——, at Niagara Falls. This patient, aged 20 years, of Dr. Clark's of that place. She had been tapped five times—52 pounds of fluid having been drawn at the last tapping. Operated May 29th, 1867. Present—Prof. Eastman, and Drs. Wetmore, Gay, Hutchings, Daggett and Potter of Buffalo, and Drs. Clark and Davis of Niagara Falls; Clark of Lockport, Warren of Lindenville, and McGary of Canada. Tumor very adherent; estimated weight 32 pounds. Time occupied 23 minutes. The pedicle was cauterized and returned into the abdomen. Vomiting first thirty-six hours, caused by chloroform. Pulse, after the operation, 112; on the sixth day it was 86. No tympany; no untoward symptoms.*

Dr. Miner said that, as the President had called for report of a recent operation for ovariotomy, he would give the main features of the case, though he had no notes of it with him, and had no intention of presenting the subject before the Society. Was much interested in the details of the operation for ovariotomy as presented by Professor White, and had no doubt that the results of the operation would depend very much upon careful observance of these points.

The question, above all others in importance, now receiving attention by surgeons in all countries, is, *what treatment shall the pedicle receive in removal of ovarian tumors?* Within a short time, the plan of applying to it, or dividing it with, the actual cautery, had come into favor. The division only of adhesive bands by this method was first practiced, and from success in this, it has been extended to the treatment of the pedicle. Results thus far, can

* July 10th, patient is almost entirely well. The operation is entirely successful.

not be said to show any gains in the proportion of recoveries over other modes of operation, yet it seems to have advantages in cases suitable for its application. It is not claimed, that it can be safely applied in all cases, or in any case where the arteries supplying the growth are large, but that it quite suffices to arrest hemorrhage in unilocular tumors and in others where the arteries are small.

He had bestowed considerable attention to the question of how best to treat the pedicle, as it appeared now the only important one connected with the operation, and he would briefly indicate his own conclusions after reporting a recent operation upon a lady in this city, wife of Mr. J. B. J., in which he was ably assisted by Drs. Burwell, Eastman, Lothrop, Wyckoff and C. F. Nichell.—The patient was 28 years old, mother of three children; was a thin, pale, timid lady, brought up and living in the luxuries of wealth. The condition of anæmia present, seemed dependent in part upon the mental anxieties inseparable from her condition, though she had suffered for the past two years very much from the presence of the growth.

The multilocular character of the tumor could be clearly discerned through the abdominal walls, and the diagnosis was as clear and positive as could be possible in any case of ovarian disease. The incision through the abdominal walls was made about three and a half inches in length while in its distended state, when contracted it was not over two and a half inches. The tumor was found but slightly adherent, except by one long band, and little difficulty was met in evacuating the numerous cysts and extracting the remaining mass from the abdominal cavity through the very small opening. The contents of the sacs varied greatly in color, consistence and quantity, and comprised in all, a large proportion of the tumor. The long, strong, adhesive band was allowed to lay by the side of the pedicle, and was divided and treated with it. The pedicle was very long, but not large, and contained two or three arteries the size of the radial or larger. The clamp was applied and division made with the actual cautery. Upon carefully opening the clamp, hemorrhage from the large arteries showed that closure was not safe or satisfactory. The pedicle being long, and being desirous of more fully testing the efficiency and safety of cauterization, the clamp was moved down upon the pedicle, and it was again subjected to thorough cauterization. Upon again relaxing the clamp the hemorrhage was as free as before, and could hardly be said to have been lessened in the trunks of the large vessels; all the small vessels were completely closed. A strong silk ligature was applied to the pedicle; the burnt edge removed entirely with the knife, and it was then left precisely as if no cauterization had been tried. It was now placed in the lower angle of the incision, and as there had been no hemorrhage or escape of the contents of the sacs into the abdominal cavity, very little sponging or other manipulation was made, and the wound was closed by twisted or hare-lip suture. Care was taken in introducing the first and second silver pins to pass them through the edges of the pedicle, just external to the ligature, so as to produce no injury to it above its point of ligation, aud also by this means to fasten it securely in its place; four silver suture pins were required and the incision was perfectly closed. A compress wetted in warm water was applied, over this, a thick, soft pad of cotton wadding, and over all, a broad bandage pinned carefully to make some pressure and afford support; this dressing was continued until recovery was complete.

After effects of operation were in no respect worthy of separate or special mention; the pulse was at no time more frequent than 88 per minute, and the constitutional disturbance comparatively slight. Opiates were not well borne; vomiting was troublesome, and pain was considerable. Recovery was complete at about the twenty-first day, and no very unfavorable symptoms were present at any time.

To return to the subject first under discussion. What disposition shall be made of the pedicle? If the vessels are large and the pedicle short, there are objections to treating it as was done in the case related; tumifaction of the abdomen might produce too much traction upon it, might even drag it from its attachment, and this plan of procedure would not be applicable to the case. It is claimed by European operators that in such cases there are no great objections to applying a ligature of metal or silk to the large vessels after the others have been closed by cauterization, and then return the pedicle into the abdominal cavity as if no ligatures had been necessary, and really, perhaps, this course is less objectionable than any other. If cauterization is sufficient, and there are not too many dangers from hemorrhage after the pedicle is returned and the circulation restored, it now seems that it has advantages over any method before practiced. If the vessels are not closed by cauterization and the length of pedicle will allow there are reasons for preferring the treatment adopted in the case of Mrs. J.

Return of the pedicle into the abdominal cavity is objectionable, mainly, for the reason that if it bleeds the hemorrhage is concealed; the crisped flesh, the ligatures, and the products of inflammation are in the cavity of the peritoneum, and are to remain, or become absorbed, or find exit by ulcerative action, all of which are in measure objectionable. If the pedicle *can* be placed, as described, in the wound, all these objections are avoided, and perhaps no others of equal importance could be urged against it. Much as has been gained in the manner of operating for ovariotomy, some of the main obstacles remain to be overcome, and it is not impossible but other discoveries may yet add to its safety.

Dr. P. H. Strong was elected to read an essay at the next regular meeting.

There were no reports offered upon prevailing diseases.

Adjourned. T. M. JOHNSON, Sec'y.

